# What barriers impede the access to healthcare toward Lesbian and Gay men: a cross-sectional study of liver transplant surgeons in China

**DOI:** 10.1080/07853890.2025.2534084

**Published:** 2025-07-21

**Authors:** Yi Xu, Ziyang Zeng, Lijuan Zhang, Minghong Sun, Yi Tao

**Affiliations:** ^a^ Department of Anesthesiology, The First Affiliated Hospital of Chongqing Medical University, Chongqing, China; ^b^ Phase I Clinical Trial Ward, The First Affiliated Hospital of Chongqing Medical University, Chongqing, China; ^c^ The First Affiliated Hospital of Chongqing University of Chinese Medicine, Chongqing, China

**Keywords:** Gay men, lesbian, liver transplant surgeons, structural equation model, the attitudes toward lesbians and gay men (ATLG)

## Abstract

**Background:**

Sexual minorities face healthcare prejudice, with clinicians often lacking knowledge of their specific needs.

**Objective:**

Investigate factors influencing Chinese liver transplant surgeons’ attitudes toward lesbians and gay men to advance health equity.

**Methods:**

.A cross-sectional web-based survey was performed in China Liver Transplantation Congress. The questionnaire included their socioeconomic characteristics, willing to receive patients with same sex orientation, the perception of lesbian and gay men, HIV cognition, previous lesbian and gay men admission history and the attitudes toward lesbians and gay men (ATLG). The structural equation model (SEM) was constructed to analyze the relationship of influencing factors of liver transplant surgeons’ attitudes toward lesbian and gay men.

**Results:**

Among 142 Chinese liver transplant surgeons, attitudes toward lesbians and gay men significantly differed by age, marital status, education, position, treatment willingness, HIV knowledge, and prior clinical exposure. Structural equation modeling demonstrated: (1) Positive HIV knowledge–treatment history association (*β* = 0.32, *p* < 0.05); (2) Socioeconomic status (*β* = 0.241, *p* = 0.01) and unawareness of MSM HIV susceptibility (*β* = 0.176, *p* = 0.04) reduced ATLG scores; (3) Prior clinical exposure predicted positive attitudes (*β* = −0.276, *p* = 0.02); (4) Lower treatment willingness associated with negative ATLG (*β* = −0.317, *p* = 0.003).

**Conclusion:**

This study elucidates the factors that influence and mediate liver transplant surgeons’ attitudes towards lesbian and gay men, as well as their willingness to treat them, with the aim of enhancing health equity for this marginalized group.

## Introduction

It is estimated that approximately 5–10% of the global population identifies as sexual minorities, including individuals who identify as gay, lesbian, bisexual, transgender, or those with gender identity disorders [1]. In China, it is estimated that sexual minorities comprise a population of no less than 70 million individuals, accounting for roughly 3–5% of the global demographic [2]. At present, significant gaps persist in China’s legal framework concerning patients with same sex orientation, the rights and well-being of this group inadequately protected against sexual orientation discrimination in medical services [3].

The sexual minorities faces many health challenges. Men who have sex with men (MSM) are more vulnerable to contracting infectious diseases, including HIV and other sexually transmitted diseases such as syphilis, Kaposi’s sarcoma (KS), viral hepatitis *etc*. Lesbians are more susceptible to HPV and Chlamydia infections, and may be at higher risk for certain communicable diseases [4]. The risk of HIV infection among MSM is approximately 19 times higher than that of the general population [5]. Furthermore, according to the minority stress hypothesis, heterosexual stigma, prejudice and discrimination would lead to long-term mental stress patients with same sex orientation, which can induce mental health problems and chronic physiological stress [3,6–8]. Studies have shown that the sexual minorities is more likely to engage in health-risk behaviors, including smoking, alcohol abuse, substance abuse, and suicide [3,9–11], who are at higher risk for adverse health outcomes such as obesity, hyperglycemia, hypertension and cardiovascular disease [12–14]. Furthermore, the minority stress hypothesis states that patients with same sex orientation would experience long-term mental stress as a result of heterosexual stigma, prejudice, and discrimination, which might result in chronic physiological stress and mental health issues. Additionally, MSM reported higher rates of cancer diagnoses, decreased rates of cancer survival, and increased headaches and incontinence. Lesbians have been linked to increased incidence of invasive breast cancer, asthma and urinary tract infections [14–16].

However, sexual minorities still encounter neglect from clinicians concerning matters related to their sexual orientation. Clinicians often lack the requisite awareness, respect, and empathy in this regard [9,17]. Ensuring the health and well-being of patients with same sex orientation has emerged as a significant public health priority. Healthcare workers are the first contacts of patients with same sex orientation and are duty-bound to provide equitable medical care [10,12]. Previous studies have demonstrated that when homosexual groups seek health care, many members experience discriminatory treatment [18]. According to a poll by Davey et al. 7% of doctors said they were uncomfortable serving gay patients, and 9% said they would stop referring patients to gay pediatricians [19]^.^ The director of the assisted reproductive technology program reported that 48% of doctors refused to treat gay couples seeking reproductive services and 17% refused to provide services to lesbian couples [20]. Studies have indicated that stigma and discrimination on sexual orientation can influence patients’ attitudes towards healthcare and may be a factor in the unequal access to healthcare [21]. Medical personnel who discriminate against homosexuality may have a variety of negative consequences, including a decrease in the group’s utilization of healthcare services [6]. Homosexuals reduce or postpone medical treatment out of fear of discrimination, which causes the illness to deteriorate or progress [18,22].

Additionally, homosexuals may hide their sexual orientation, which will influence doctors’ judgment of their health risks and treatment decisions, such as lowering the frequency of HIV testing, immunizing against HPV, and preventing other sexually transmitted diseases [18,23–25].

During surgical operations, surgeons frequently encounter situations where they come into direct contact with patients’ blood. HIV transmission can take place through contamination of surgical instruments or when a surgeon accidentally incises their hand, thereby exposing themselves to a patient’s blood. Organ transplant surgeons, in particular, face a heightened level of occupational exposure to infectious diseases like HIV, which may significantly impact their willingness to offer medical services. Previous research has indicated that transplant surgeons are more inclined to perform organ transplants for patients infected with hepatitis B virus (HBV) or hepatitis C virus (HCV) than for those living with HIV. When juxtaposed with HBV or HCV infections, surgeons exhibit a greater degree of apprehension towards HIV infection. They perceive HIV infection as having a higher likelihood of disrupting their personal lives. The fear of contracting HIV during surgery acts as a deterrent, reducing surgeons’ willingness to carry out organ transplantation procedures for HIV-positive patients [26]. China’s current Regulation on Human Organ Transplantation makes no explicit provisions regarding the donation or transplantation of organs involving individuals living with HIV [27]. Meanwhile, the lack of explicit anti-discrimination policies, combined with traditional gender norms, may intensify the stigma associated with such cases during clinical decision making processes. This stigma has the potential to influence surgical assessments and patient care strategies [28,29].

In this study, we posit that liver transplant surgeons, as a distinct subgroup within the broader surgeon population, are more susceptible to HIV contamination. We maintain that investigating the attitudes of liver transplant surgeons towards patients with same sex orientation could offer insights into potential barriers that hinder this group’s access to healthcare services. Gaining a comprehensive understanding of these attitudes is of paramount importance for addressing healthcare disparities and ensuring the equitable delivery of healthcare. To identify the potential factors influencing liver transplant surgeons’ attitudes and their willingness to accept patients with same sex orientation, as well as to provide empirical evidence for improving health equity among this population, we conducted a nationwide survey of liver transplant surgeons in China, thus to delve deeper into their attitudes towards patients with same sex orientation.

## Methods

### Study design

Between 14 and 20 August 2022, we conducted a cross-sectional survey by using non-probability sampling method in China Liver Transplantation Congress. Surgeons are requested to complete a questionnaire on the network platform(This is a classic questionnaire, refers to supplement 1 for details). To be eligible, participants needed to fill more than 95% of the questionnaire items.

### Ethical considerations

This study was approved by the Ethics Committee of the First Affiliated Hospital of Chongqing Medical University (No. 2022-73). The surgeons were all told that the surveys would be anonymous, and participation in this study was completely optional. Furthermore, there was no financial reward for participants’ time dedicated, but their participation would make a great contribution to the healthcare career. Participants were assured that their information would only be used for this study, and the answers to questionnaires would only be utilized for this study. Our procedures were carried out in accordance with the Declaration of Helsinki.

### Informed consent statement

Informed consent was obtained from all participants and the form was in writing.

### Measures

Six measures of the questionnaire were included as follows:

1) Socioeconomic characteristics were collected including age (year), marriage (single, married), annual income (ten thousand) and positions (resident doctor, visiting staff, professor). 2) Willing to receive patients with same sex orientation (‘0’ means No, ‘1’ means Yes). 3) Whether MSM is susceptible to HIV (‘0’ means No, ‘1’ means Yes). 4) HIV cognition included ‘knowing the phases of AIDS’, ‘knowing the route of HIV transmission’, ‘knowing AIDS prevention measures’ and ‘knowing AIDS high-risk group’. In the process of evaluation, ‘0’means answered wrongly, ‘1’ means answered correctly. 5) Previous homosexuality admission history included ‘treated patients with HIV’ and ‘have done liver transplant surgery for homosexuality’. In the process of evaluation, ‘0’ had no homosexuality admission history, ‘1’ means had received homosexual patients. 6) The attitudes toward lesbians and gay men (ATLG) scale (Herek, 1988) was translated, which can be divided into two subscales for separate assessment of attitudes toward lesbians and gay men [16]. The scale has 2 dimensions and 20 items, including the attitudes toward lesbians (10 items) and the attitudes toward gay men (10 items), which used Likert’s 5-grade scoring method, that is, from ‘strongly disagree’ to ‘strongly agree’, the scale was scored from ‘1’ point to ‘5’ points, with higher scores indicating more negative attitudes.

### Statistical analysis

We applied descriptive statistics for the surgeons and the attitude toward the lesbian and gay men. Categorical data were presented as number (frequencies), continuous variables were presented as means and SDs if normally distributed and medians and IQRs if not, groups were compared with two independent sample *t* test, one-way ANOVA or Kruskal–Wallis test. Pearson correction analyses were taken to explore the correlation of factors and ALTG. The structural equation model (SEM) was adopted to analyze the relationship among factors, The SEM model fit criteria used were, root mean square error of approximation (RMSEA) <0.08, comparative fit index (CFI) >0.90, Tucker–Lewis index (TLI)>0.90 [17]. All tests performed by two-sides test, with *p*<.05 as the statistical difference. SPSS 27.0 was applied to analyze the data, the SEM was performed through Amos 24.0.

## Results

### Factors and surgeons’ attitudes toward lesbian and gay men

A total of 142 liver transplantation surgeons participated this survey. Table 1 shows surgeons’ socioeconomic characteristics and their attitudes toward lesbians and gay men. The average age of the surgeons was 27.9 ± 6.9 years old. The average years of working was 4.8(Q1–Q3: 29–33). In attitudes toward lesbian and gay men, the results showed statistical differences in age, marriage, education, ‘willing to receive homosexual patients’, ‘knowing the route of HIV transmission’, and ‘knowing AIDS high-risk group’. In the dimensions of attitudes toward gay men, the results showed statistical differences in age, marriage, annual income, ‘willing to receive homosexual patients’ and ‘have done liver transplant surgery for homosexuality’. In the dimensions of attitudes toward lesbians, the results showed statistical differences in marriage, education, ‘willing to receive homosexual patients’ and ‘have done liver transplant surgery for homosexuality’.

**Table 1.** Univariate analysis of the factors and surgeons’ attitudes toward lesbians and gay men (*N* = 142).

Variables	n (%)	attitudes toward lesbians (x ± s)	*P*	attitudes toward gay men (x ± s)	*P*	attitudes toward homosexual (x ± s)	*P*
Age			.10		.01		.02
≤25	51(36)	21.3 ± 7.3		23.3 ± 8.9		44.5 ± 15.3	
26–30	43(30)	22.1 ± 8.0		27.1 ± 10.4		49.3 ± 16.4	
31–35	31(22)	21.3 ± 7.9		27.5 ± 8.9		48.8 ± 10.7	
>35	17(12)	25.7 ± 7.0		30.4 ± 5.3		56.1 ± 14.5	
Marriage			.02		<.001		.001
Single	78(55)	20.9 ± 7.5		23.9 ± 9.7		44.9 ± 15.7	
married	64(45)	23.9 ± 7.6		29.3 ± 7.7		53.2 ± 13.3	
Education			<.001		.21		.005
Junior college	42(30)	29.3 ± 7.3		23.1 ± 7.8		52.4 ± 13.2	
Undergraduate	43(30)	28.4 ± 9.8		23.6 ± 7.5		52.0 ± 14.8	
Master degree	26(18)	24.1 ± 9.5		21.4 ± 8.0		45.5 ± 16.2	
Doctor degree	31(22)	21.4 ± 8.3		20.1 ± 7.2		41.5 ± 15.0	
Position			.65		.15		.26
resident doctor	91(64)	21.9 ± 7.7		25.4 ± 9.5		47.3 ± 15.9	
visiting staff	44(31)	22.6 ± 8.5		27.6 ± 9.3		50.2 ± 15.1	
professor	7(5)	24.2 ± 3.3		30.5 ± 2.2		54.7 ± 4.6	
Years of working			.09		.85		.47
≤3	78(55)	26.8 ± 9.0		22.1 ± 7.8		48.9 ± 15.3	
3–5	18(13)	30.3 ± 8.4		22.8 ± 6.7		53.1 ± 11.1	
5–10	23(16)	23.5 ± 7.6		23.3 ± 7.4		46.8 ± 14.6	
≥10	23(16)	24.7 ± 11.1		21.4 ± 8.4		46.1 ± 18.0	
Annual income			.37		.02		.06
≤10	93(65)	21.6 ± 7.8		24.8 ± 10.0		46.4 ± 16.2	
10–20	39(27)	23.3 ± 7.7		29.2 ± 7.1		52.5 ± 13.0	
≥20	10(7)	24.3 ± 5.3		28.9 ± 4.7		53.1 ± 9.4	
Willing to receive homosexual patients			.002		<.001		.02
No	26(18)	58.7 ± 7.9		32.0 ± 9.0		25.4 ± 7.7	
Yes	116(82)	53.6 ± 7.3		25.1 ± 8.8		21.6 ± 7.5	
Whether MSM is susceptible to HIV			.07		.01		.58
No	16(11)	51.5 ± 6.6		21.6 ± 7.0		21.4 ± 6.6	
Yes	126(89)	54.9 ± 7.7		27.0 ± 9.3		22.4 ± 7.8	
Knowing the phases of AIDS			.81		.72		.70
No	135(95)	54.5 ± 7.6		26.4 ± 9.2		22.3 ± 7.6	
Yes	7(5)	55.3 ± 8.4		25.1 ± 8.9		21 ± 8.5	
Knowing the route of HIV transmission			.25		.11		.02
No	39(27)	55.7 ± 7.1		28.2 ± 8.1		24.4 ± 6.3	
Yes	103(73)	54.1 ± 7.8		25.7 ± 9.5		21.5 ± 8.0	
Knowing AIDS prevention measures			0.91		0.90		0.42
No	27(19)	54.3 ± 9.0		26.2 ± 7.7		23.3 ± 7.5	
Yes	115(81)	54.5 ± 7.3		26.4 ± 9.5		22.0 ± 7.7	
Knowing AIDS high-risk group			.56		.36		.02
No	16(11)	54.8 ± 8.1		27.0 ± 8.7		23.6 ± 7.4	
Yes	126(89)	54.1 ± 6.9		25.5 ± 9.9		20.5 ± 7.7	
Treated patients with HIV			.20		.10		.81
No	99(70)	54.0 ± 7.7		25.5 ± 9.1		22.4 ± 7.8	
Yes	43(30)	55.7 ± 7.5		28.3 ± 9.3		22.1 ± 7.3	
Have done liver transplant surgery for lesbian and gay men			.01		.06		,87
No	131(92)	55.0 ± 7.4		26.8 ± 9.2		22.3 ± 7.7	
Yes	11(8)	48.5 ± 8.6		21.3 ± 8.5		21.9 ± 7.2	

Data were expressed as *n* (%), mean ± SD. Statistical analysis was performed using the two independent sample *t* test and one-way ANOVA. All tests were performed by two-sides test, with *p* < 0.05 as the statistical difference.

### Correlations between factors and attitudes toward the lesbian and gay men

In Table 2, Positive attitude of liver transplantation surgeons toward lesbian and gay men was correlated with younger age, unmarried status, lower education and position, higher cognition of HIV, willing to receive homosexual patients and ever received homosexual patients. Only a small difference revealed in positive attitude toward lesbians and gay men.

**Table 2.** The correlation between factors and the attitudes toward lesbians and gay men (*N* = 142).

Factors	attitudes toward lesbians (x ± s)	attitudes toward gay men (x ± s)	attitudes toward lesbian and gay men (x ± s)
Age	0.163*	0.268*	0.244*
Marriage	0.195*	0.291*	0.274*
Education	−0.157*	−0.338*	−0.283*
Position	−0.119	−0.210*	−0.187*
Years of working	−0.004	−0.117	−0.072
Annual income	0.045	0.196*	0.123
Willing to receive homosexual patients	−0.190*	−0.291*	−0.272*
Whether MSM is susceptible to HIV	−0.042	0.191*	0.133
Knowing the phases of AIDS	−0.038	−0.300	−0.038
Knowing the route of HIV transmission	−0.174*	−1.250	−0.163*
Knowing AIDS prevention measures	−0.067	−0.100	−0.028
Knowing AIDS high-risk group	−0.204*	−0.079	−0.151*
Treated patients with HIV	−0.323*	−0.301*	−0.344*
Have done liver transplant surgery for homosexuality	−0.014	−0.161	−0.104

Pearson correction analyses were applied to explore the correlation of factors and ALTG (higher scores indicating more negative attitudes). All tests were performed by two-sides test, with *p*<.05 as the statistical difference.

* means *p*<.05.

MSM: Men Who Have Sex With Men.

HIV: Human Immunodeficiency Virus.

AIDS: Acquired Immune Deficiency Syndrome.

**Figure 1.**
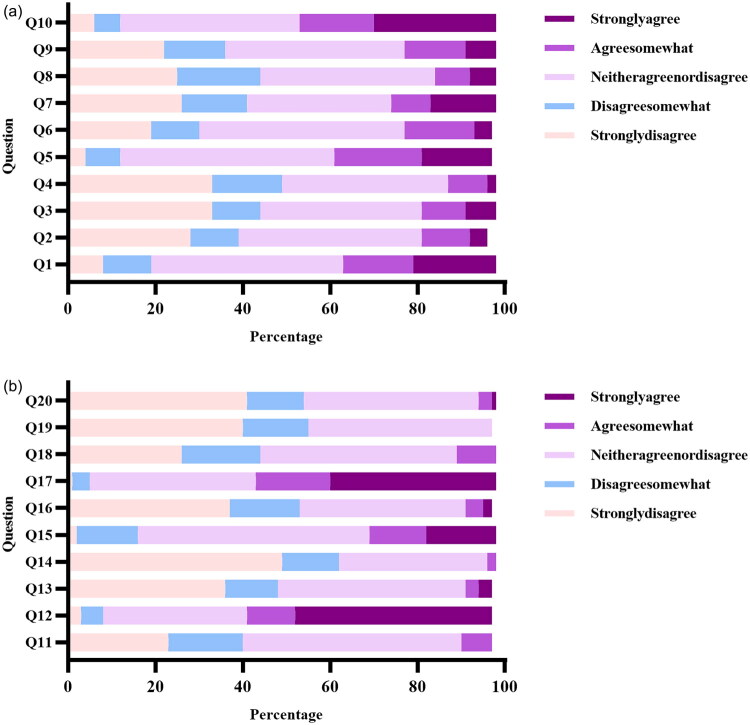
(A) Comparisons of the attitudes of liver transplantation surgeons toward gay man (*N* = 142) (Q1: Male homosexual couples should be allowed to adopt children the same as heterosexual couples; Q2: I think male lesbian and gay men are disgusting; Q3: Male lesbian and gay men should not be allowed to teach school; Q4: Male lesbian and gay men is a perversion; Q5: Just as in other species, male lesbian and gay men is a natural expression of sexuality in human men; Q6: If a man has homosexual feelings, he should do everything he can to overcome them; Q7: I would not be too upset if I learned that my son were a homosexual; Q8: Homosexual behavior between two men is just plain wrong; Q9: the idea of male homosexual marriages seems ridiculous to me; Q10: Male lesbian and gay men is merely a different kind of lifestyle that should not be condemned.). (B) Comparisons of the attitudes of liver transplantation surgeons toward lesbians (*N* = 142) (Q11: Lesbians just can’t fit into our society; Q12: a woman’s lesbian and gay men should not be a cause for job discrimination in any situation; Q13: Female lesbian and gay men is detrimental to society because it breaks down the natural divisions between the sexes; Q14: State laws regulating private, consenting lesbian behavior should be loosened; Q15: Female lesbian and gay men is a sin; Q16: the growing number of lesbians indicates a decline in social morals; Q17: Female lesbian and gay men in itself is no problem, but what society makes of it can be a problem; Q18: Female lesbian and gay men is a threat to many of our basic social institutions; Q19: Female lesbian and gay men is an inferior form of sexuality; Q20:Lesbians are sick.).

Comparisons of the attitudes of liver transplantation surgeons toward gay men and lesbians were shown in Figure 1. The percentage of strongly disagree toward gay man was 20.78%, neither agree nor disagree was 41.69%, and 11.20% was strongly agree. The percentage of strongly disagree toward lesbians was 26.41%, neither agree nor disagree was 41.97% and 10.99% was strongly agree. Differences were not existed in attitudes of liver transplantation surgeons toward gay man and lesbians (X^2^=0.07, *p*=.96).

### Structural equation model

**Figure 2.**
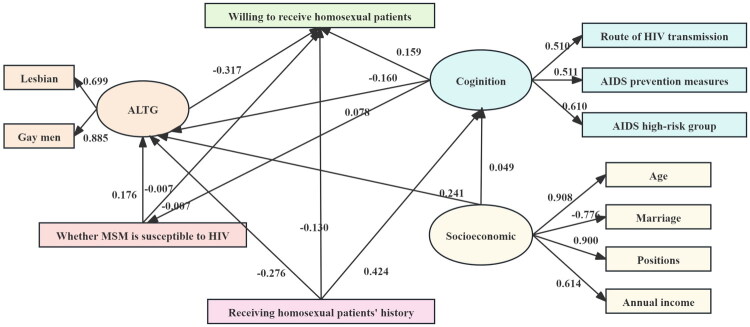
Structural equation model with path coefficients (β). ATLG: The Attitudes Toward Lesbians and Gay Men Scale; MSM: Men who have sex with men; HIV: Human immunodeficiency virus; AIDS: Acquired immunodeficiency syndrome

The structural equation model extracted six factors 11 items. The model showed that cognition of HIV, socioeconomic status, receiving homosexual patients’ history and whether MSM is susceptible to HIV influenced ALTG. ALTG, whether MSM is susceptible to HIV, previous homosexual patients admission history and cognition of HIV influenced willing to receive homosexual patients. Moreover, socioeconomic status and receiving homosexual patients’ history may as direct or indirect factors influence willing to receive homosexual patients. The results showed the model had a good fit and satisfied recommended standard (RMSEA = 0.039, CFI = 0.980, and TLI = 0.971) (Figure 2).

In the structural equation model, treated history of lesbian and gay men was positively related to HIV cognition ((β = 0.424, *p*<.001). Higher socioeconomic status (β = 0.241, *p*=.01) and unknown MSM is susceptible to HIV (β = 0.176, *p*=.04) had negatively impacts on ATLG. Receiving homosexual patients’ history (β = –0.276, *p* = .02) had positive attitudes on homosexual patients. ATLG was negatively correlated with willing to treat homosexual patients (β = –0.317, *p* = .003). Parameter estimation of the structural equation model is shown in Table 3.

**Table 3.** Parameters estimation of structural equation model.

Influencing factors	unstandardized estimates	Standard error	Critical ratio	standardized estimates	*P*
Receiving homosexual patients’ history→cognition	0.261	0.079	3.321	0.424	<0.001
Socioeconomics→ATLG^4^	0.203	0.081	2.520	0.241	.01
receiving homosexual patients’ history →ATLG	−2.980	1.219	−2.445	−0.276	.02
Whether MSM is susceptible to HIV→ATLG	2.950	1.459	2.022	0.176	.04
ATLG→Willing to receive homosexual patients	−0.023	0.008	−3.002	−0.317	.003

ATLG: the attitudes toward lesbians and gay men (higher scores indicating more negative attitudes).

HIV: Human Immunodeficiency Virus.

## Discussion

### Principal results

In order to give focused action to promote the health equity of patients with same sex orientation, this study investigated the factors influencing and mediating liver transplant doctors’ attitudes toward patients with same sex orientation and willingness to treat them in China. We conducted a cross-sectional web-based survey in China Liver Transplantation Congress. Structural equation model can express mediational linkages, and is beneficial for evaluating intricate theoretical models with many variables and relationships. For considering liver transplant surgeons are at high risk of HIV acquisition, we supposed their attitudes toward patients with same sex orientation could reflect barriers impede the access to healthcare. Our findings revealed that ALTG is influenced by socioeconomic position, cognitive ability, and homosexuality treatment history. These factors may also have a direct or indirect impact on a surgeon’s willingness to accept homosexual patients.

### Comparison with prior work

Regarding socioeconomic characteristics, we found that the older the surgeons were, the more prejudiced they were against lesbian and gay men. This finding may have resulted from older groups’ acceptance of traditional gender roles and preconceived notions about sexual orientation [18,20]. Surgeons in married status showed more negative attitudes than single ones. Chinese traditional society promotes people to get married and have children, which may have an impact on social attitudes regarding lesbian and gay men, especially among those who have already married [21]. Doctors with higher education levels showed a more positive attitude towards lesbian and gay men, which was in line with previous research results, a survey in Dutch revealed that residents with greater levels of education were also more tolerant [21]. While education has been shown to alter people’s values, better educated surgeons were more knowledgeable and inclined to pursue their careers, it can also foster higher cognitive complexity and complicated reasoning, which empowers people to see lesbian and gay men more objectively.

In Chinese culture, men were typically associated with traits like courage, strength, and confidence. Lesbian and gay men was seen as a danger to the gender structure, which resulted in strongly held negative opinions. It was previously recognized that gay men were more likely to contract HIV, and that prejudice against gay men would always be greater than prejudice against lesbians [30,31]. It’s possible that the surgeons in higher positions had more tolerant views toward lesbian and gay men since they were more skilled and knowledgeable. Surgeons are knowledgeable about the HIV transmission method and AIDS high-risk group tended to have more positive attitudes toward lesbians. Furthermore, gender has been shown to be related to attitudes in earlier research [32,33], which was not included in our analysis, as the majority of the liver transplant surgeons who participated in this study were male.

Results from the structural equation model suggested that surgeons with lesbian and gay men treating experience have lower level of discrimination and are more accepting of gay men and lesbians, which was consistent with the study by Smith et al. [18]. Studies have found that people with more comprehensive knowledge of HIV tend to have more positive attitudes towards lesbian and gay men [34]. Ignorance of lesbian and gay men will lead to discrimination or prejudice for lesbian and gay men [32]. In terms of this issue, we proposed that medical schools take into account granting medical students access to the sexual minority, as this could potentially lessen the prejudice that future healthcare professionals will face towards this community. Bristols et al. noted that medical staff members’ cultural competency and readiness to treat the sexual minority can be enhanced by knowledge training on sexual orientation and gender identity [26]. Moreover, surgeons with favorable attitudes toward lesbians and gay men were more ready to receive patients with same sex orientation, namely practice makes perfect.

In addition, there exists an interaction between policies and doctors’ attitudes. While the current regulations in China do not explicitly bar HIV infected individuals from undergoing transplants, in practical clinical settings, doctors may decline to perform surgeries on MSM patients. This refusal often stems from ‘risk aversion’ or ‘divergent policy interpretations’. To a certain extent, the ambiguity of the regulations may exacerbate clinical biases. Clear policy guidance can play a positive role in regulating the attitudes of relevant medical professionals [35]. We advocate for the introduction of detailed laws and regulations that clearly define ‘HIV infection status’ as the assessment criterion for transplantation, rather than ‘sexual orientation’. This would help reduce institutional level discrimination. Although the HOPE Act aims to enhance organ access for people living with HIV, due to the ineffective implementation of the policy, the number of organs actually procured from HIV positive deceased donors remains far below initial expectations [36]. Therefore, our future work will focus on promoting the rationalization of policies and the updating of doctors’ clinical guidelines. This endeavor is not only a requirement of medical ethics and social equity but also contributes to improving the overall landscape of public health.

### Proposals

In view of the aforementioned findings, we made the following specific recommendations to enhance the health equity of the sexual minority. First and foremost, it is imperative to eliminate discriminatory laws. Although China’s laws and policies concerning sexual minorities are continuously evolving and making progress, there remains significant scope for further advancement. Secondly, efforts should be made to enhance surgeons’ knowledge of HIV and sexually transmitted diseases (STDs). To bridge these knowledge gaps, medical education programs ought to incorporate practical sessions and courses covering topics like sexual health education, cultural diversity, mental health, and STDs. Additionally, organizing discussion forums can serve as an effective platform for students to express their opinions and emotions, thereby fostering an environment of education and tolerance. Finally, doctors should strive to gain a deeper understanding of lesbian, gay, bisexual, and transgender (LGBT) individuals and treat them in a scientific and objective manner. Hospitals can also organize more free clinic sessions specifically for LGBT individuals. Moreover, we recommend implementing community engaged initiatives to deliver targeted healthcare services. These could include free infectious disease testing and free condom distribution, with the aim of reducing the overall incidence of infectious diseases at the grass roots level.

## Limitations

Although this study identified obstacles that hinder lesbian and gay men from accessing healthcare, it also exhibits several notable limitations. Firstly, we employed a net based cross sectional survey, which are conducted at a specific time point or within a defined period and scope, are unable to establish causal relationships between variables. Secondly, the sample size of this study is generally inadequate for conducting subgroup analyses to thoroughly validate the findings. In future research, we intend to incorporate a wider array of stratified characteristics and increase the sample size to enrich our research outcomes. Although some studies have demonstrated significant gender based differences in surgeons’ attitudes toward lesbian and gay men, gender was not included as a variable in this study. This is because the majority of liver transplant surgeons are male. Lastly, the use of non-probability sampling in our study may restrict the generalizability of the results to other regions or medical contexts. Given the specific nature of the target population in this survey, who constitute a highly specialized and limited group. Our future research plans encompass expanding the sample scope and adopting stratified sampling to enhance the external validity of our findings.

## Conclusions

This study developed a model to identify the factors influencing Chinese liver transplant surgeons’ attitudes toward lesbian women and gay men, which is of utmost significance for advancing health equity and promoting the well-being of sexual minorities. Our findings indicated that surgeons’ knowledge of HIV, their socioeconomic status, and their prior experience of caring for patients with same sex orientation all have an impact on their attitudes toward lesbian women and gay men. With the goal of enhancing health equity for this sexual minority group, we suggest that the first and most pivotal step is to eliminate discriminatory regulations. Following this, it is imperative to enhance doctors’ proficiency in HIV and other sexually transmitted diseases (STDs). By doing so, healthcare providers will be better equipped to offer appropriate care and education. Finally, community-focused initiatives ought to be implemented to deliver specialized healthcare services, which has the potential to significantly decrease the hidden incidence of infectious diseases among the sexual minority community.

## Data Availability

The data sets generated during and/or analyzed during this study are available from the corresponding author on reasonable request.
